# Supplementary material for: High-Fat Diet Enhances the Liver Metastasis Potential of Colorectal Cancer through Microbiota Dysbiosis
Source: Cancers (Basel). 2022 May 24;14(11):2573. doi: 10.3390/cancers14112573 (PMC9179364; doi:10.3390/cancers14112573)
Supplement: Supplementary file 1 [file cancers-14-02573-s001.zip › cancers-1708122-supplementary.pdf]

Rats:

Table S1丨Primers used for the quantitative real-time PCR.

TNF-α	F:5′ CCACGCTCTTCTGTCTACTG 3'<br>R:5′ GCTACGGGCTTGTCACTC 3′
IL-1β	F:5′ TGTGATGTTCCCATTAGAC 3'<br>R:5′ AATACCACTTGTTGGCTTA 3′
OCLN	F: 5′ GTTTACTGGCAGAACTCG 3'<br>R: 5′ ATAGTCTCCCACCATCCT 3′
ZO-1	F: 5′ GATGAGCGGGCTACCTTA 3'<br>R: 5′ ATGGGAGCGAACTGAATG 3′
MMP2	F: 5′ CTGGGCAACAAGTATGAGA 3'<br>R: 5′ GCTGCCACAAGGAATAGG 3′
MMP9	F: 5′ GACAGCTCCTCCCACTATGT 3'<br>R: 5′ TCAGCAATGGCATCAAAA 3′
CXCL12	F:5′ GCATCAGTGACGGTAAGC 3'<br>R:5′ AGGGCACAGTTTGGAGTGTTGAGGA 3′
TLR4	F:5′TGCTGCCAACATCATCCA3'<br>R: 5′GAGCGGCTACTCAGAAACTGCCATG3′
IL-6	F: 5′ GAGTTCCGTTTCTACCTG 3'<br>R: 5′ CTTAGCCACTCCTTCTGT 3′
Fibronectin	F:5′ TCGCTTTGACTTCACCAC 3'<br>R:5′ CTTCCTCGCTCAGTTCGT 3′

Mice：

Table S2丨Primers used for the quantitative real-time PCR.

TNF-α	F:5′ CAGGCGGTGCCTATGTCTC 3'<br>R:5′ CGATCACCCCGAAGTTCAGTAG 3′
IL-1β	F:5′ GAAATGCCACCTTTTGACAGTG 3'<br>R:5′ TGGATGCTCTCATCAGGACAG 3′
OCLN	F: 5′ TGAAAGTCCACCTCCTTACAGA 3'<br>R: 5′ CCGGATAAAAAGAGTACGCTGG 3′
ZO-1	F: 5′ GAGCGGGCTACCTTACTGAAC 3'<br>R: 5′ GTCATCTCTTTCCGAGGCATTAG 3′
MMP2	F: 5′ ACCTGAACACTTTCTATGGCTG 3'<br>R: 5′ CTTCCGCATGGTCTCGATG 3′
MMP9	F: 5′ GCAGAGGCATACTTGTACCG 3'<br>R: 5′ TGATGTTATGATGGTCCCACTTG 3′
CXCL12	F: 5′ TGCATCAGTGACGGTAAACCA 3'<br>R: 5′ CACAGTTTGGAGTGTTGAGGAT 3′
TLR4	F: 5′ GCCTTTCAGGGAATTAAGCTCC 3'<br>R: 5′ GATCAACCGATGGACGTGTAAA 3′
IL-6	F: 5′ CTGCAAGAGACTTCCATCCAG 3'<br>R: 5′ AGTGGTATAGACAGGTCTGTTGG 3′
DSV	F:5′AGTTTGATCMTGGCTCAG3′<br>R:5′GGTTACCTTGTTACGACTT3′
